# Correction: Structural characterization of two nanobodies targeting the ligand-binding pocket of human Arc

**DOI:** 10.1371/journal.pone.0321004

**Published:** 2025-03-25

**Authors:** José M. Godoy Muñoz, Lasse Neset, Sigurbjörn Markússon, Sarah Weber, Oda C. Krokengen, Aleksi Sutinen, Eleni Christakou, Andrea J. Lopez, Hongyu Zhang, Clive R. Bramham, Petri Kursula

Hongyu Zhang is not included in the author byline. Hongyu Zhang should be listed as the ninth author and affiliated with Department of Biomedicine, University of Bergen, Bergen, Norway. The contributions of this author are as follows: conceptualization, methodology, resources, and supervision.
