# Supplementary figures and images for: Structural characterization of two nanobodies targeting the ligand-binding pocket of human Arc
Source: PLoS One. 2024 Apr 29;19(4):e0300453. doi: 10.1371/journal.pone.0300453 (PMC11057775; doi:10.1371/journal.pone.0300453)

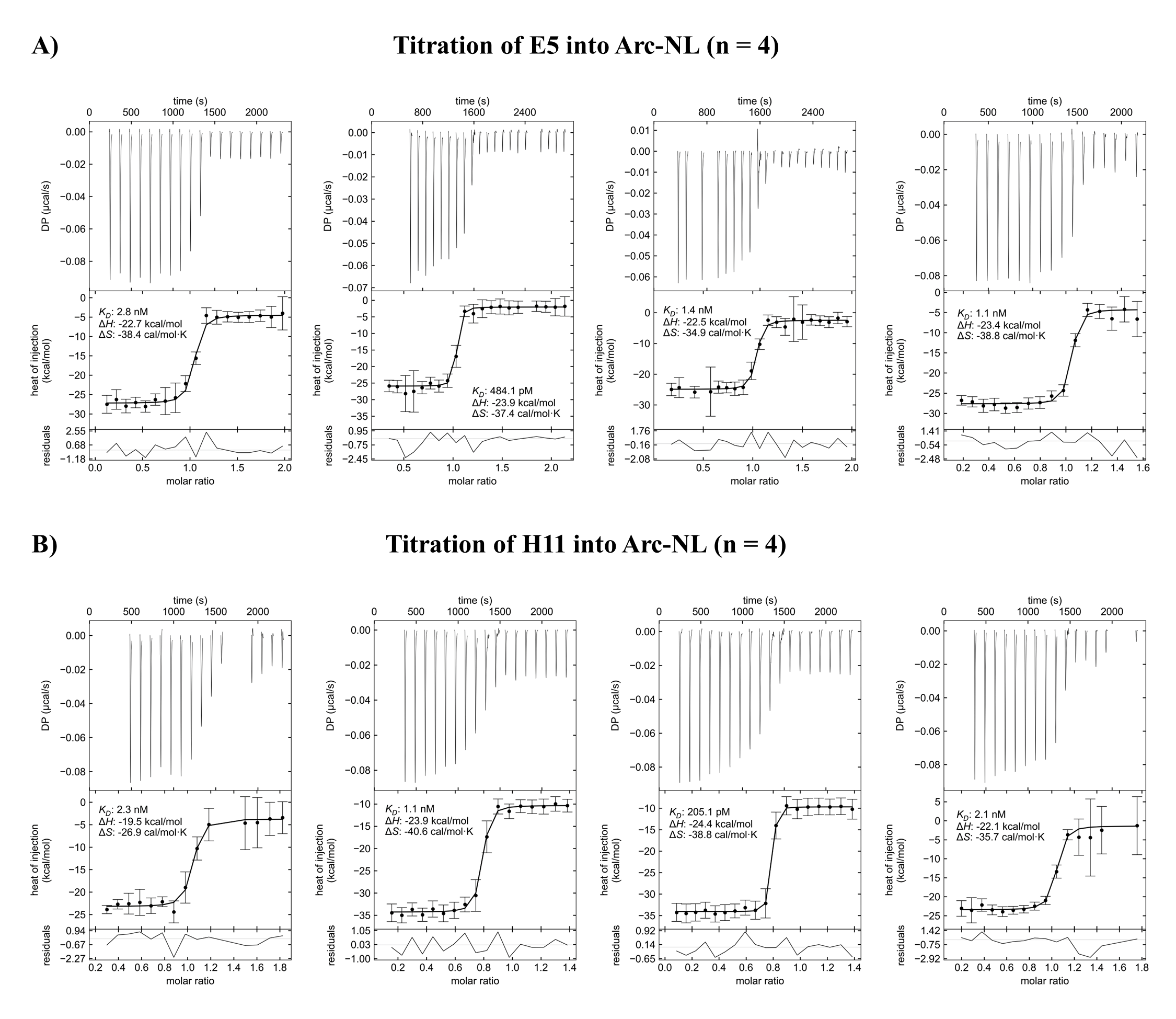

Supplement: S1 Fig — (TIF) [file pone.0300453.s001.tif]
