# Supplementary material for: Structural characterization of two nanobodies targeting the ligand-binding pocket of human Arc
Source: PLoS One. 2024 Apr 29;19(4):e0300453. doi: 10.1371/journal.pone.0300453 (PMC11057775; doi:10.1371/journal.pone.0300453)
Supplement: S2 Fig — (PDF) [file pone.0300453.s002.pdf]

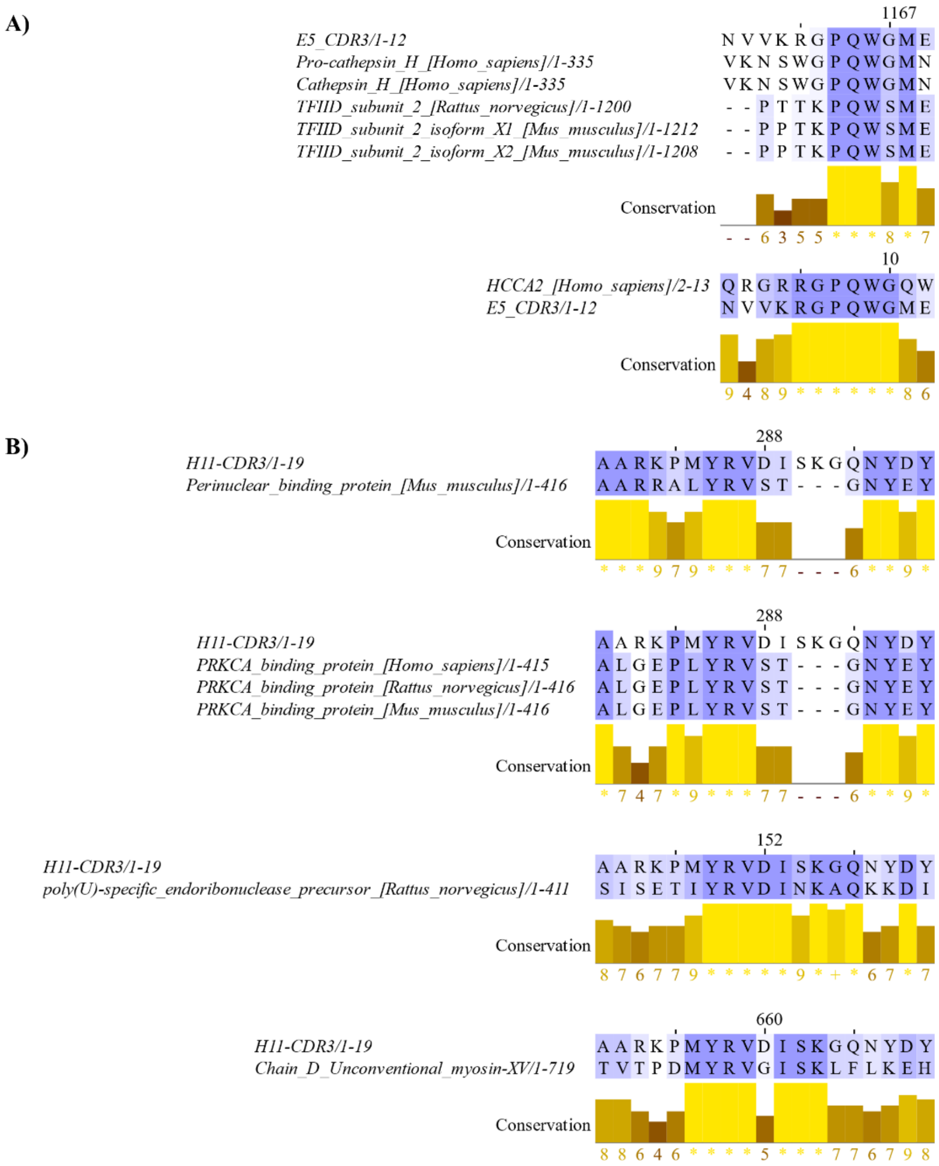

S2 Fig. Multiple sequence alignment using Blast hits for E5 and H11 CDR3 loops. (A) Multiple sequence alignment using Clustal Omega [61] of the E5 CDR3 with the protein sequences obtained in Blast [60]. (B) Pairwise alignment performed with Clustal Omega of the H11 CDR3 with the protein sequences acquired from Blast. The data were visualized with Jalview.
