# Supplementary material for: Structural characterization of two nanobodies targeting the ligand-binding pocket of human Arc
Source: PLoS One. 2024 Apr 29;19(4):e0300453. doi: 10.1371/journal.pone.0300453 (PMC11057775; doi:10.1371/journal.pone.0300453)
Supplement: S1 Table — (PDF) [file pone.0300453.s003.pdf]

***S1 Table. Protein sequence matches for the CDR3s of E5 and H11 provided by Blast**. For each sequence, its corresponding protein, species, E-value and ID are shown. Relevant matches (E < 10) are highlighted in bold. Abbreviations: HCCA2, hepatocellular carcinoma-associated protein 2; PRKCA, protein kinase C alpha; TFIID, transcription factor II D.*

**Query**	**Protein match**	**Species**	**E-value**	**Sequence ID**
**E5-CDR3**	Pro-cathepsin H	*Homo sapiens*	3.4	6CZK (PDB)
	Cathepsin H	*Homo sapiens*	3.4	AAH02479.1
	HCCA2	*Homo sapiens*	28	BAB62266.1
	TFIID – Subunit 2	*Rattus norvegicus*	81	NP_579853.1
	TFIID – Subunit 2 (Isoforms X1 and X2)	*Mus musculus*	81	XP_011243959.1
**H11-CDR3**	Perinuclear binding protein	*Mus musculus*	0.98	CAA86675.1
	Poly(U)-specific endoribonuclease precursor	*Rattus norvegicus*	16	NP_001177998.1
	Unconventional myosin XV	*Mus musculus*	16	7UDT (PDB)
	PRKCA-binding protein	*Homo sapiens*	31	NP_001034672.1
	PRKCA-binding protein	*Rattus norvegicus*	31	NP_445912.2
	PRKCA-binding protein	*Mus musculus*	31	NP_001039023.1
